# The effectiveness of a family-centred intervention after traumatic brain injury: A pragmatic randomised controlled trial

**DOI:** 10.1177/02692155211010369

**Published:** 2021-04-15

**Authors:** Mari S Rasmussen, Nada Andelic, Are H Pripp, Tonje H Nordenmark, Helene L Soberg

**Affiliations:** 1 Department of Physical Medicine and Rehabilitation, Oslo University Hospital, Oslo, Norway; 2 Institute of Health and Society, Research Centre for Habilitation and Rehabilitation Models & Services (CHARM), Faculty of Medicine, University of Oslo, Oslo, Norway; 3 Oslo Centre of Biostatistics and Epidemiology Research Support Services, Oslo University Hospital, Oslo, Norway; 4 Department of Psychology, University of Oslo, Oslo, Norway; 5 Department of Physiotherapy, Faculty of Health Sciences, OsloMet – Oslo Metropolitan University, Oslo, Norway

**Keywords:** Traumatic brain injury, rehabilitation interventions, family, health related quality of life, randomised controlled trial

## Abstract

**Objectives:**

To determine the effectiveness of a family-centred intervention for patients with traumatic brain injury and family members.

**Design:**

Open-labelled, two-armed randomised controlled trial.

**Settings:**

Outpatient clinic and family residences.

**Participants:**

Sixty-one patients (33 women) with traumatic brain injury, with mean (SD) age 43.8 (12.2), and 63 family members (33 women), with mean (SD) age 42.6 (11.3), were assign to intervention (*n* = 30 families) and control group (*n* = 31 families).

**Intervention:**

An eight-session single-family intervention to improve individual and family functioning.

**Outcome measures:**

Self-reported questionnaires at start-of-treatment, median (IQR) 11.4 (8.4, 15.9) months post-injury, and at two follow-ups, 2.7 (2.3, 3.8) and 9.2 (8.2, 9.9) months after start-of-treatment. Primary outcome measures were the SF-36 Mental Component Summary (MCS) and Caregiver Burden Scale (CGB). Secondary outcome measures were the Family Adaptability and Cohesion Evaluation Scale (FACES) and Quality of Life after Brain Injury Questionnaire (QOLIBRI). Group differences were analysed with linear mixed-model analysis for repeated measurements.

**Results:**

No significant between-group differences were found. The intervention group significantly improved on the MCS, the CGB and FACES in the treatment period, whereas the controls did not. The mean (SD) MCS change in the treatment period was 2.4 (1.1) points *P* = 0.028 in the intervention group. Mean (SE) MCS scores were 47.9 (1.26) and 47.3 (1.27) in the intervention and control group at last follow-up.

**Conclusions:**

Receiving an eight-session family intervention, in addition to specialised rehabilitation for the patients, was not superior to rehabilitation at a specialised traumatic brain injury outpatient clinic.

## Introduction

In Europe, the incidence of traumatic brain injury is approximately 2.5 million each year.^1^ Such injuries may have long-lasting consequences that not only have an impact upon the patients but also upon the patient’s family and friends.^2^ Cognitive, emotional and behavioural changes in the injured person disrupt the family dynamics and lead to persistent, unhealthy family functioning in a significant proportion of families after traumatic brain injury.^3,4^ Diminished health-related quality of life and increased levels of psychological distress are reported by both patients^5,6^ and family members.^7^ Increased and persistent levels of caregiver burden in the family members^8^ might reduce their capacity to care for the injured family member and negatively affect the patient’s recovery.^9^

Consequently, interventions to help support the family have been suggested^10^ and have been researched, for example, in relation to patients who have suffered a stroke.^11^ The family serves as the primary support system for patients, and interpersonal relationships are recognised as an important factor influencing all aspects of the rehabilitation process.^12^ Despite this, rehabilitation after traumatic brain injury has often been individually oriented, and family members have been treated as passive actors in the process.^13^

Few studies have evaluated the effectiveness of family system interventions, and most study results draw on information from either the patients or the caregiver.^14^ Moreover, findings on family functioning are often not reported, and it has been emphasised that studies should report on both patient and caregiver outcomes because the family is a unit.^14^ The current evidence for family interventions after traumatic brain injury are limited by low sample sizes as well as poor fidelity and randomisation techniques.^14,15^ Much uncertainty still exists about the effectiveness of family-centred interventions on patients and caregivers or family members, and there is a need for studies evaluating such interventions.

In a small pilot study, Stevens et al.^16^ examined the effectiveness of a family intervention specifically developed for families facing traumatic brain injury or spinal cord injury. Based on family systems theory, the intervention included elements from solution-focused therapy and cognitive behavioural therapy. The authors reported improvements in caregiver burden, psychological distress and problem solving strategies, and recommended further studies of the intervention’s effect on outcomes such as quality of life and family functioning.^16^ Based on the results of the pilot study by Stevens et al., we conducted a feasibility study prior to the current study.^17^ The objective of the present study was to determine the effectiveness of this family intervention for patients with traumatic brain injury and their family members. We hypothesised that there would be significant improvements in the family intervention group for mental health-related quality of life, family functioning, communication and satisfaction in patients and family members as well as reduced caregiver burden over time for the family members when compared to the control group. Further, we wanted to explore within-group changes in outcomes during the treatment period.

## Methods and materials

The Regional Committee for Medical and Health Research Ethics, South-East Norway (#2016/1215) and the Data Protection Officer at Oslo University Hospital approved the study. The study was registered in ClinicalTrials.gov with the identification number NCT03000400 and reported according to the Consolidated Standards of Reporting Trials (CONSORT) guidelines.^18^ Oral and written informed consent were obtained from all participants in the study.

This was an open-labelled, two-armed randomised controlled trial conducted at Oslo University Hospital, Norway, in collaboration with a municipal health care service. Enrolment of families took place from January 2017 to June 2019. The study population included patients admitted to the outpatient clinic for rehabilitation after mild-to-severe traumatic brain injury. The patients had been hospitalised for observation/rehabilitation in the acute phase or were referred by their general practitioner in case of persistent post-concussion symptoms. Patients were screened for eligibility upon admission by a physiatrist. The included patients selected family members for participation. Family members were defined as any relative, including spouses, partners, parents, children, or others actively involved in the patient’s daily life.

Inclusion criteria for the patients were the following: (a) age between 16 and 65 years; (b) diagnosed with traumatic brain injury of any severity according to the International Classification of Diseases (ICD-10) system (S06.0-S06.9); (c) a Rancho Los Amigos Revised Scale score of 8^19^; (d) traumatic brain injury sustained 6 to 18 months ago; and (e) home dwelling. Inclusion criteria for family members were the following: (a) age between 18 and 65 years; and (b) being actively involved in the patient’s daily life, with weekly contact.

Patients and family members were excluded in the following cases: (a) inability to speak/read Norwegian; (b) a pre-injury learning disability; (c) an ICD-10 diagnosis of severe psychiatric or degenerative neurological illness; (d) on-going substance abuse; and (e) having other family members who required professional care.

The families were randomised (1:1) according to a computer generated list with random block sizes of 4 to 8. An independent researcher was responsible for the randomisation process, and first-author MSR contacted the patients and provided information about the group allocation. Blinding the participants and the rehabilitation professionals to the allocation was not possible, but the data were entered and managed with a coded group allocation in the database by an independent research assistant, and the allocation code was not broken until the primary analyses of data from the first to the last follow-up were conducted.

The data collection was administered by an independent research assistant that was blinded with regard to group allocation. The participants answered self-reported outcome measures at three different times: at start-of-treatment, at two-month follow-up (after completion of the family intervention) and at eight-month follow-up after start-of-treatment, with parallel time points for the control group. As the first assessment time point took place after randomisation, it is not per definition a true baseline. Hence, we defined the first assessment as start-of-treatment. The families allocated to the intervention group answered the questionnaires at start-of-treatment and at the two-month follow-up as part of the first and last sessions of the family intervention, whereas the families allocated to the control group received the questionnaires by mail, supplied with an information/instruction letter. At the eight-month follow-up, all families received the questionnaires by mail and were offered a final consultation with a physiatrist at the outpatient clinic.

Sociodemographic data were collected by a short questionnaire developed by authors MSR and HLS at the start-of-treatment and included age, gender, marital status, kinship to the injured person, whether family members live in the same household as the patients, number of people in the household, level of education (dichotomised as low/high with high representing college/university degree), patients’ pre-injury employment status, current employment status and patients’ self-reported comorbidities. Injury-related variables were obtained from medical records, including time since injury, injury mechanism, neuroimaging results of intracranial injury, length of hospital stay, Glasgow Coma Scale score,^20^ and the Head Abbreviated Injury Score (AIS).^21^ Post-concussion symptom pressure was registered at first follow-up using the Rivermead Post-Concussion Symptoms Questionnaire.^22^

The selection of outcome measures was based on constructs targeted in the intervention,^16^ and recommended outcome measures in traumatic brain injury research.^23^ The primary outcome measures were the following:

- Mental health related quality of life was assessed with the Mental Component Summary, which is a sum score based on the mental health subscales on the 36-item Short-Form Health Survey (SF-36).^24^
- The Caregiver Burden Scale, which is a multidimensional scale that assesses caregivers’ perceived subjective burden within five dimensions: general strain, isolation, disappointment, emotional involvement and environment.^25^

The secondary outcome measures were:

- The Family Adaptability and Cohesion Evaluation Scale, fourth edition, was used to assess level of cohesion and flexibility in the family or couple system.^26^ For research purposes, a circumplex ratio score ranging from 0 (worst) to 10 (best) is recommended. A score ⩾1 indicates balanced levels of cohesion and flexibility in the system. In addition, 10 items assess the level of family communication with the Family Communication Scale, and 10 items assess the level of family satisfaction along the Family Satisfaction Scale.^26^
- The Quality of Life after Brain Injury Questionnaire, a condition specific measure designed specifically to assess quality of life after traumatic brain injury, was applied to the patients.^27^ It consists of six subscales that include four satisfaction scales (cognition, self, daily life and autonomy) and two bothered scales (emotions and physical problems).

All patients in the study received rehabilitation at the specialised outpatient clinic, which comprised a clinical examination and, if needed, services by a multidisciplinary team consisting of five different health professionals. The aim of the rehabilitation was to assist the patients’ return to daily life activities and work by providing information, support and recommendations. The outpatient clinic treatment is described in more detail in a study by Howe et al.^28^ In the control group, the family members were invited to attend a 2.5-hour psychoeducational group session conducted by an occupational therapist and a psychologist from the multidisciplinary team. The group session focused on brain anatomy, traumatic brain injury and post-injury challenges in functioning and in resuming daily life activities and work, but not specifically on family functioning.

In the intervention group, the rehabilitation at the outpatient clinic was supplied with the Traumatic Brain Injury/Spinal Cord Injury Family Intervention.^16^ This theoretically based family intervention comprises eight 90-minute sessions focusing on specific topics. The intervention manual has appeared as supplementary material in a previous publication.^16^ The aims described in the manual were to improve patients’ and family members’ individual functioning and the family functioning and to enact positive changes in communication, level of conflict, family satisfaction and interpersonal boundaries.^16^ Some minor cultural adjustments to fit the Norwegian context were made in advance of the randomised controlled trial and are described in a previous publication.^17^

To each family separately, the main-group facilitator (author MSR, physical therapist) delivered the sessions according to the instruction manual, with approximately one session per week. The sessions comprised both theoretical and practical components and had a fixed structure but were individually tailored to accommodate each family’s unique needs. In the sessions, participants were given the opportunity to share personal experiences and family challenges relevant to their specific situation. Handouts and between-session tasks were provided, and the families were encouraged to apply the learned skills and techniques to real life situations. Based on the families’ needs and preferences, the intervention was delivered at the Oslo University Hospital, in the family’s home, or in appropriate municipal premises. The group-facilitator scheduled the sessions based on the families’ availability. For 10 of the families in the intervention group, a rehabilitation professional (nurse or occupational therapist) from the collaborating municipality attended as co-facilitator. Table 1 provides an overview of the intervention topics.

**Table 1.** Overview of intervention topics.^16^

Session	Topic	Content
1	Introduction	Information about the study. Introduction and overview of expectations and completion of start-of-treatment questionnaires.
2	Making meaning	Extracting beliefs and experiences related to traumatic brain injury.
3	Shifting focus	Positive changes after traumatic brain injury. Understanding the relationship between thoughts, feelings and behaviour.
4	Managing emotions	Physiological changes when emotions escalate. Recognising ‘warning signs’ of emotional escalations. Strategies for overcoming negative emotions.
5	Communicating effectively	Fighting fairly. Communication danger signs. Strategies for effective communication.
6	Finding solutions	Moving from a problem-oriented to solution-oriented perspective. Formulating useful goals. Problem-solving skills.
7	Boundary making	Externalising the problems. Education on healthy versus unhealthy family dynamics. Importance of self-care.
8	Summarising and farewell	Summary of skills learned, feedback from the participants and completion of two-months follow-up questionnaires.

All group-facilitators had received training in the intervention, and they participated in a feasibility study of the family intervention.^17^ Furthermore, elements from a publication by Winter et al.^29^ were used to assess the main group-facilitator’s adherence to the intervention manual and administration of the family intervention. The elements measuring task completion included the following: (a) explained purpose of each session clearly; (b) used appropriate pace and language; (c) showed sensitivity to the participant responses; (d) responded clearly to participants’ questions, (e) demonstrated overall fidelity to the Traumatic Brain Injury/Spinal Cord Injury Family System Intervention manual; and (f) explained next step of intervention. The fidelity items were rated as poor, good, or excellent by a municipal health professional after completion of the family intervention for nine (30%) of the families in the intervention group. All items concerning fidelity were rated as excellent by the municipal health professional.

Additionally, in the last session of the family intervention, participants were asked to rate the level of satisfaction with the sessions and of satisfaction with the group-facilitator’s delivery of the sessions on a numeric scale ranging from 0 (not at all satisfied) to 10 (very satisfied). The participants were very satisfied with the intervention sessions (mean score of 9.3; SD 0.9) and with the way the sessions were delivered by the group-facilitator(s) (mean score of 9.6; SD 0.6).

## Statistical methods

Data were analysed with Stata 16 and with an intention-to-treat approach, including all subjects randomised regardless of group, compliance with treatment, or withdrawals. Descriptive statistics were used to describe the study population, and demographic variables were compared using χ^2^, Mann-Whitney U tests, or t-tests, as appropriate. Continuous variables were presented as mean and standard deviation (SD) or median and interquartile range (IQR), and categorical variables were presented as frequency and percentage.

Sample size was determined based on the primary outcomes. For the Mental Component Summary on the SF-36, the study on patients with moderate to severe traumatic brain injury by Andelic et al.^30^ was used, and we inserted 44 points (SD12) with a difference of 5 points between the groups. With a power of 80% and a significance level of 0.05, the predicted sample size was 66 patients, with 33 families in each arm of the randomised controlled trial. In addition, we estimated that there would be two family members per patient. And for the Caregiver Burden Scale, Manskow et al.s’^31^ study on Norwegian caregivers of persons with severe traumatic brain injury was used. A reduction of 0.4 points on the Caregiver Burden Scale is equal to a moderate effect size, and the power calculation yielded a sample size of 126 caregivers.

To evaluate the intervention effect, a linear mixed model analysis for repeated measurements with a random intercept was conducted to investigate between-group differences at start-of-treatment and at the two- and eight-month follow-ups. The main effect of treatment, the main effect of time and the interaction term between treatment and time were applied as fixed effects in the statistical model. Random effects were the subjects. Results are presented as mean differences with 95% confidence intervals (CIs) for all three assessment time points. All tests were two-sided and assumed a significance level of *P* = 0.05. Assumptions of all statistical tests were not violated.

## Results

**Figure 1.**
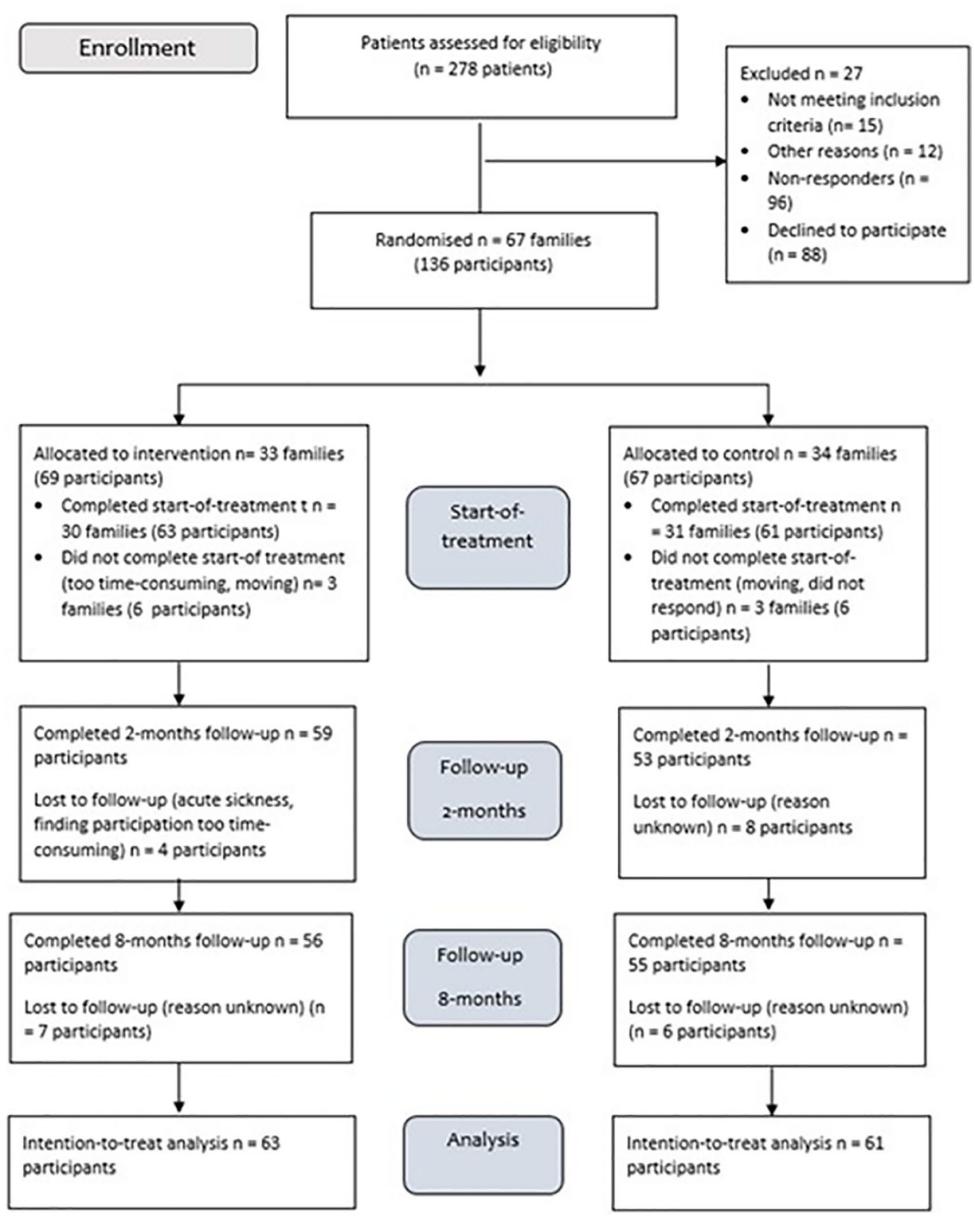
CONSORT flow chart.

Figure 1 shows the flow chart for study recruitment and retention flow. Of the 251 eligible patients, 67 patients and 69 family members consented to participation and were randomised to the intervention group (*n* = 33 families) and control group (*n* = 34 families). Before the assessment at the start-of-treatment, six families (8.8 %) withdrew. Data from at least one time point were available for 124 participants (91%). In four families, the two first or last sessions were combined into one session pursuant to the families’ request to minimise use of time. The families were recruited approximately one year post-injury. Median (IQR) in months from start-of-treatment to two months’ follow-up was 2.7 (2.3, 3.8) months and 9.2 (8.2, 9.9) months to the eight-month follow-up. No adverse effects were reported during the study.

Participant characteristics and injury-related data are displayed in Table 2. There were no significant differences in characteristics or outcome measures at start-of-treatment between the groups. Most patients (82%) had a mild traumatic brain injury and reported persistent post-concussion symptoms as assessed by the Rivermead Post-concussion Questionnaire. Almost all family members (92%) were the patients’ spouse/partner.

**Table 2.** Sample characteristics of the intention-to-treat population at start-of-treatment (*n* = 124) in personal factors, living arrangements and injury-related factors.

Variables	Intervention		Control	
	*n* = 63 participants		*n* = 61 participants	
	Patients (*n* = 30)	Family members (*n* = 33)	Patients (*n* = 31)	Family members (*n* = 30)
Age, years (mean, SD)	45.0 (11.8)	43.5 (12.2)	42.6 (10.3)	41.6 (10.0)
Female sex, *n* (%)	15 (50.0)	18 (54.5)	18 (58.1)	15 (50.0)
Married/cohabitating, *n* (%)	29 (96.7)	32 (97.0)	30 (96.8)	29 (96.7)
Kinship to the patient				
Spouse/partner, *n* (%)		29 (87.9)		29 (96.7)
Parent, *n* (%)		1 (3.0)		1 (3.3)
Children, *n* (%)		3 (9.1)		
Length of relationship in years				
<1 year, *n* (%)	3 (10.3)	3 (9.4)		
1–5 years, *n* (%)	3 (10.3)	4 (12.5)	4 (13.3)	4 (13.8)
>5 years, *n* (%)	23 (79.3)	25 (78.1)	26 (86.7)	25 (86.2)
Living with injured person, *n* (%)		28 (84.8)		29 (96.7)
Number of family members in the insured’s household, mean (range)	3.0 (0–6)		3.1 (1–6)	
Level of education				
Low, *n* (%)	9 (30.0)	9 (27.3)	7 (22.6)	6 (20.0)
High, *n* (%)	21 (70.0)	24 (72.7)	24 (77.4)	24 (80.0)
Employment status				
Preinjury				
Employed/studying, *n* (%)	27 (90.0)		30 (96.8)	
Preinjury not employed, *n* (%)	3 (10)		1 (3.2)	
Post-injury				
Employed/studying, *n* (%)	5 (16.7)	27 (81.8)		26 (86.7)
Partly sick-leaved, *n* (%)	12 (40.0)	1 (3.0)	21 (67.7)	3 (10.0)
Sick-leaved 100%, *n* (%)	13 (43.3)	5 (15.2)	10 (32.3)	1 (3.3)
Injury characteristics				
Time since injury months, median (IQR)	11.4 (8.3, 15.3)		11.4 (8.5, 16.8)	
GCS, median (IQR)	15 (11.8, 15.0)		15 (14.0, 15.0)	
AIS, median (IQR)	2 (2.0, 3.3)		1 (1.0, 2.0)	
Findings on CT/MRI, *n* (%)	11 (36.7)		7 (22.6)	
Falls, *n* (%)	11 (36.7)		12 (38.7)	
Traffic accidents, *n* (%)	10 (33.3)		9 (29.0)	
Mechanical object, *n* (%)	6 (20.0)		8 (25.8)	
Violence, *n* (%)	1 (3.3)		1 (3.2)	
Others, *n* (%)	2 (6.7)		1 (3.2)	
RPQ (*n* = 56), mean (SD)	29.9 (10.9)		25.8 (10.9)	
Self-reported comorbidities, *n* (%)	6 (20.0)		5 (16.1)	

GCS: Glasgow Coma Scale Score; AIS: abbreviated injury scale score; CT/MRI: computed tomography/magnetic resonance imaging; RPQ: Rivermead Post-concussion Questionnaire; IQR: interquartile range; SD: standard deviation.

**Figure 2.**
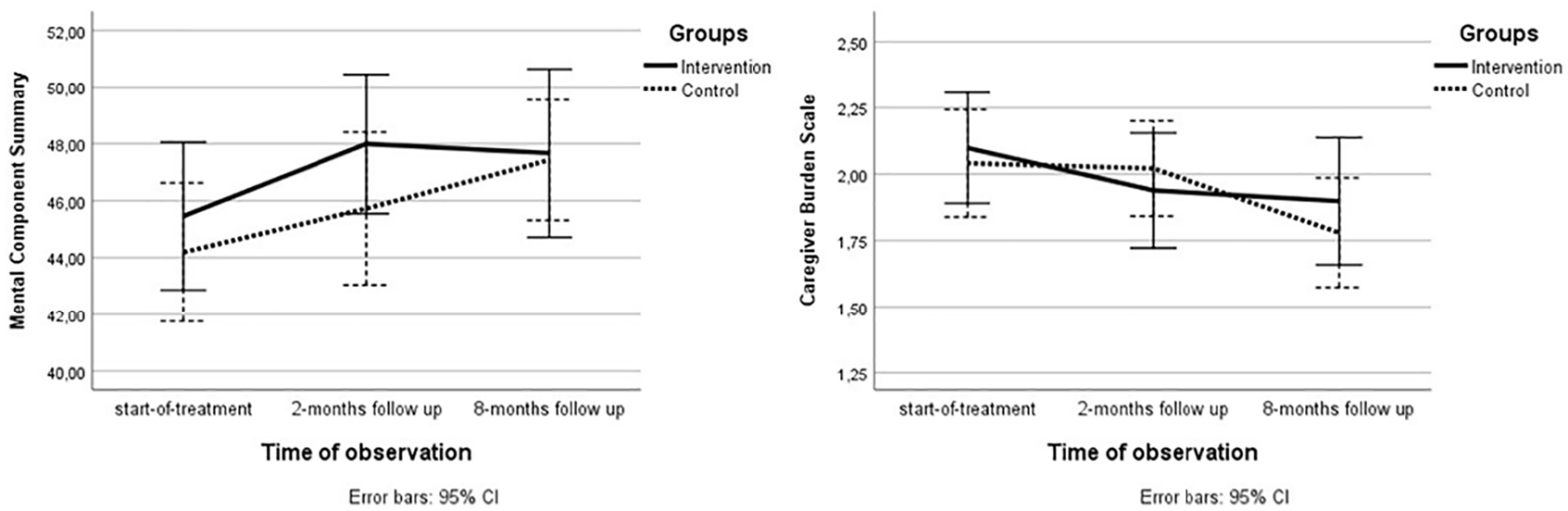
Graphical presentation of mean scores on the primary outcome measures, the mental component summary and the caregiver burden scale, per group on start-of-treatment, two-months follow up and eight-months follow up.

Results from the multilevel model analysis with between-group mean differences are displayed in Table 3. There were no significant between-group differences on the primary outcome measures, the Mental Component Summary and the Caregiver Burden Scale, at the follow-ups. However, there were significant within-group improvements on the Mental Component Summary (*P* = 0.028) and the Caregiver Burden Scale (*P* = 0.003) from start-of-treatment to two-month follow-up in the intervention group. Mental health related quality of life and level of caregiver burden improved over time in both groups (Figure 2).

**Table 3.** Mean difference for each outcome between the groups (control compared with intervention) at start-of-treatment, follow-up two-months and follow-up eight-months using a linear mixed model for repeated measurements.

Measure	Mean difference (95% CI) between groups, start of treatment	*P*-value	Mean difference (95% CI) between groups, two-months follow-up	*P*-value	Mean difference (95% CI) between groups, eight-months follow-up	*P*-value
MCS	−1.27 (−4.67 to 2.13)	0.464	−1.93 (−5.43 to 1.57)	0.280	−0.61 (−4.11 to 2.90)	0.734
CGB	−0.01 (−0.34 to 0.21)	0.632	0.07 (−0.21 to 0.34)	0.633	−0.01 (−0.29 to 0.26)	0.920
FACES	0.15 (−0.25 to 0.55)	0.470	0.03 (−0.38 to 0.21)	0.871	0.15 (−0.26 to 0.56)	0.482
FCS	4.36 (−0.26 to 0.56)	0.331	−1.81 (−4.42 to 13.14)	0.692	2.75 (−10.77 to 7.14)	0.548
FSS	3.47 (−6.49 to 13.43)	0.495	−0.19 (−10.37 to 10.00)	0.972	1.13 (−9.09 to 11.35)	0.828
QOLIBRI	−2.23 (−10.58 to 6.12)	0.601	0.88 (−7.59 to 9.35)	0.838	1.10 (−7.37 to 9.56)	0.799

MCS: mental component summary; CGB: caregiver burden scale; FACES: family adaptability and cohesion evaluation scale; FCS: family communication scale; FSS: family satisfaction scale; QOLIBRI: quality of life after brain injury questionnaire.

No significant between-group differences were demonstrated on the secondary outcome measures, the Family Adaptability and Cohesion Evaluation Scale and the Quality of Life after Brain Injury Questionnaire, at two or eight months. At all assessment time points, both groups reported balanced levels of cohesion and flexibility in the family system, indicated by a mean circumplex ratio score >1, as well as high levels of family communication with mean scores on the Family Communication Scale >62 percentiles.^26^

**Figure 3.**
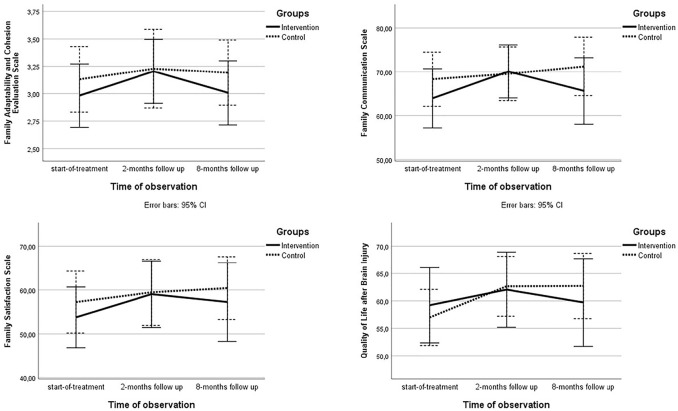
Graphical presentation of mean scores on the secondary outcome measures, the family adaptability and cohesion evaluation scale, the family communication scale and the family satisfaction scale, per group on start-of-treatment, two-months follow up and eight-months follow up.

From start-of-treatment to the two-month follow-up, the intervention group had significant improvements in family functioning on the circumplex ratio score (*P* = 0.027), Family Communication Scale (*P* = 0.002) and Family Satisfaction Scale (*P* = 0.030), whereas the control group did not. The patients in both groups had a mean score <60 points on the Quality of Life after Brain Injury Questionnaire at start-of-treatment, indicating reduced quality of life.^32^ However, they improved over time, whereas only the patients in the control group had a significant change from start-of-treatment to two-month follow-up (*P* = 0.002) (Figure 3). Within group changes are displayed in Table 4.

**Table 4.** Mean scores on primary and secondary outcomes at all assessment time points with within-group changes from start-of-treatment to follow-up two-months and follow-up eight-months, by group.

	Intervention group					Control group				
	Start-of-treatment *n* = 63	Two-months follow-up *n* = 59	Eight-months follow-up *n* = 56	Change from start-of-treatment to follow-up two-months	Change from start-of-treatment to follow-up eight-months	Start-of-treatment *n* = 61	Two-months follow-up *n* = 53	Eight-months follow-up *n* = 55	Change from start-of-treatment to follow-up two-months	Change from start-of-treatment to follow-up eight-months
	Mean (SE)	Mean (SE)	Mean (SE)			Mean (SE)	Mean (SE)	Mean (SE)		
MCS (0–100 worst-best)	45.5 (1.21)	47.9 (1.24)	47.9 (1.26)	2.4*	2.4*	44.2 (1.24)	46.0 (1.29)	47.3 (1.27)	1.8	3.1*
CGB (1–4 best-worst)	2.1 (0.10)	1.9 (0.10)	1.8 (0.10)	−0.2*	−0.3	2.0 (0.10)	2.0 (0.10)	1.8 (0.10)	0.0	−0.2*
FACES (0–10 worst-best)	3.0 (0.14)	3.2 (0.15)	3.0 (0.15)	0.2*	0.0	3.1 (0.15)	3.2 (0.15)	3.2 (0.15)	0.1	0.1
FCS (10–99 worst-best)	63.9 (3.14)	70.7 (3.18)	66.7 (3.22)	6.8*	2.8	68.3 (3.19)	68.9 (3.28)	69.5 (3.26)	0.6	1.2
FSS (10–99 worst-best)	53.8 (3.56)	59.5 (3.62)	58.1 (3.67)	5.7*	4.3	57.2 (3.62)	59.3 (3.73)	59.2 (3.70)	2.1	2.0
QOLIBRI (0–100 worst-best)	59.2 (3.01)	62.2 (3.05)	61.0 (3.06)	3.0	1.8	57.0 (3.01)	63.1 (3.06)	62.1 (3.04)	6.1*	5.1*

MCS: mental component summary; CGB: caregiver burden scale; FACES: family adaptability and cohesion evaluation scale; FCS: family communication scale; FSS: family satisfaction scale; QOLIBRI: quality of life after brain injury questionnaire.

* Significant within-group changes *P* < 0.05.

## Discussion

Contrary to our hypothesis, this clinical trial showed no extra benefit of the eight-session family intervention in addition to ordinary rehabilitation at a specialised outpatient clinic on mental health related quality of life, condition-specific quality of life, caregiver burden and family functioning (including communication and satisfaction). However, in the intervention period, there were statistically significant improvements in mental health related quality of life, caregiver burden, family functioning, communication and satisfaction in the intervention group, indicating that the family intervention possibly contributed with a boost in the recovery process.

Our results differed from those reported in the randomised pilot study of the Traumatic Brain Injury/Spinal Cord Injury Family Intervention on eight individuals with spinal cord injury and their family members, which demonstrated significant reductions in psychological distress, burden and improved problem-solving skills in favour of the intervention group. However, this pilot study was small in size and underpinned the need for further examination of the intervention’s effectiveness.^16^ Both traumatic brain injury and spinal cord injury are conditions that can induce stress on the family system and its functioning.^16,33^ However, changes in cognitive and behavioural functioning represent well-known disabilities after sustaining a traumatic brain injury that are less common after spinal cord injury. Moreover, contextual factors, such as access to formal health services were different from those in the present study, making direct comparison of the results difficult.

Adopting elements from the family therapy field, with a theoretical foundation in family systems theory, is recommended in the traumatic brain injury literature as it enables systemic changes in the family unit.^10^ However, few interventions have worked with patients and caregivers as active participants together and do not report on outcomes that reflect the family or dyadic health as a whole, such as family functioning.^14^ In general, comparing family intervention studies after traumatic brain injury is challenging because study aims, methodology as well as outcome measures differ among the studies.^14,15^

In the present study, both groups showed improvement over time in mental health related quality of life, and the family members’ subjective caregiver burden was reduced in the follow-up period. This is in contrast to findings in a study on Norwegian caregivers of persons with severe traumatic brain injury, which reported increased caregiver burden two years after the injury.^8^ However, most patients in the present study had consequences following mild traumatic brain injury. Although they experienced persistent symptoms, the recovery is generally good for persons with mild traumatic brain injury, and function improves over time.^34^ Thus, it might be that the level of subjective caregiver burden also improved over time for the majority of family members in the present study. Furthermore, the optimal timing for delivery of family interventions is uncertain,^14^ and might be questioned in the present study as well. The rationale for delivering the intervention from 6 to 18 months post-injury was to ensure that the participants had experienced family challenges related to the injury before receiving the intervention as most patients at this point have been discharged to their homes and families.

If the quality of standard care or ‘treatment as usual’ is comprehensive in randomised controlled trial control groups, the effect sizes might be reduced.^35^ The treatment provided to the control group might have influenced the results in this study, as all patients received the specialised rehabilitation. The family members in the control group also attended the educational group session about traumatic brain injury. Because of this, families in both groups may have experienced that many of their needs were met through specialised outpatient rehabilitation. Moreover, completing the self-reported questionnaires might have opened up discussions about family functioning and communication for those in the control group. This is an issue also highlighted in a randomised controlled trial of a patient intervention after stroke, in which some control group participants reported that the assessments promoted reflection, adjustments and help-seeking behaviour.^36^

The mean level of family functioning showed balanced levels of cohesion and flexibility in the family system at start-of-treatment. Thus, a selection bias is possible, related to those who volunteered to participate in this study. It might be that families with problematic dynamics and more troubled family functioning, who could have benefitted more from the intervention, found it too difficult to address family issues in addition to coping with the traumatic brain injury. When people voluntarily participate in studies, the researcher cannot be sure whether the participants are those most in need of the intervention.^37^

Further, participation in the intervention asked families to attend eight sessions and complete home tasks, and four families asked to combine sessions to reduce use of time. Based on the experiences from the feasibility study conducted before commencement of the randomised controlled trial, a pragmatic approach was considered necessary to succeed with the intervention delivery.^17^ In addition, pragmatic adjustments to each respective family is embedded in the intervention sessions. Feasibility of delivery mode of caregiver and dyad interventions is emphasised in both the stroke and traumatic brain injury literature, as finding time to attend several sessions can be challenging for family members due to busy everyday life schedules.^11,14^ Thus, this was an effectiveness rather than an efficacy trial, where the pragmatic context and adjustments might have diluted the power to detect differences in the study arms but strengthened the external validity of the study.

Moreover, the patients in this study reported condition-specific quality of life just above the suggested cut-off for poor quality of life at the two- and eight-month follow-ups.^32^ Whereas the patients in the control group showed a significant improvement on this outcome, the intervention group did not. With symptoms commonly experienced after traumatic brain injury, such as fatigue, headache and poor concentration,^34^ attending eight family sessions might be perceived as a burden rather than an opportunity. An abridged version of the family intervention, adjusted to topics provided as part of the specialised rehabilitation process, might have been more appropriate for this patient group.

Strengths of this study were that we followed the Consolidated Standards of Reporting Trials (CONSORT) statement^18^ and that feasibility with regard to delivery of the intervention and outcome assessments were evaluated prior to the full-scale randomised controlled trial.^17^ Furthermore, the use of pragmatic elements makes this study relevant to clinical practice. To our knowledge, this is one of the first family-centred intervention studies with an emphasis on patients with mild traumatic brain injury with a protracted course of recovery.

In addition to the methodological challenges that have been raised in the discussion, the study has some limitations that should be noticed. All participants were recruited from the same specialised outpatient clinic, and the same therapist was responsible for delivering the intervention to all families. Hence, our results may not be generalisable to outpatient clinics with different structures and processes of care. Although we did succeed in recruiting a sufficient number of patients needed in this study, we included fewer family members than estimated, most likely due to cultural factors, such as the typical Norwegian family structure. Further, we had to end the inclusion period after 2.5 years due to the project’s time frame.

Several outcome measures were used in this study, as recommended when evaluating the effectiveness of complex interventions.^38^ However, recovery after traumatic brain injury is multifaceted, and we cannot be sure we chose the right outcomes to capture the intervention’s actual impact. Furthermore, it could be considered a limitation that the families did not need to meet a clinical threshold for family functioning to be included in the study, and future studies should aim to deliver the family intervention to families with more troubled family functioning. Also, this manualised intervention did not allow for more extended individualised treatment targeting specific issues. Because most patients had sustained a mild traumatic brain injury, precaution should be made when generalising the results to families facing more severe injuries.

Any illness and disability can put stress on the family unit, and the Traumatic Brain Injury/Spinal Cord Injury Family Intervention could be applied to families dealing with illness and disability in general.^16^ However, many intervention studies focus on a specific condition.^14^ With regard to generalisability, future studies should consider including participants with different conditions, such as stroke or traumatic brain injury, or other chronic neurologic conditions, as the families might experience many of the same needs.^14^ This could improve transfer of knowledge between different health sectors. Additionally, this is relevant with regard to implementation of interventions in municipal health care settings, as they are less specialised and provide services and support to persons with various conditions and their families. Moreover, planning intervention studies with a mixed methods approach, such as combining quantitative measures with semi-structured interviews, may help to define key components of interventions and should be considered in future research.

From this study we conclude that receiving the theoretically based family intervention, in addition to outpatient specialised rehabilitation for patients with traumatic brain injury and their family members, was not superior to only receiving outpatient specialised rehabilitation in improving mental health related quality of life, condition-specific quality of life, caregiver burden and family functioning after traumatic brain injury. However, our findings imply that the family intervention might have contributed to a boost in individual and family functioning during the intervention period.

**Clinical message**

- Receiving a theoretically based eight-session family intervention, in addition to specialised rehabilitation at a traumatic brain injury outpatient clinic, was not superior to only receiving specialised rehabilitation in improving individual and family functioning in patients with mild-to-severe traumatic brain injury and their family members.
